# Skin Conductance Response to the Pain of Others Predicts Later Costly Helping

**DOI:** 10.1371/journal.pone.0022759

**Published:** 2011-08-03

**Authors:** Grit Hein, Claus Lamm, Christian Brodbeck, Tania Singer

**Affiliations:** 1 Laboratory for Social and Neural Systems Research, Department of Economics, University of Zurich, Zurich, Switzerland; 2 Social, Cognitive and Affective Neuroscience Unit, Faculty of Psychology, University of Vienna, Vienna, Austria; 3 Department of Psychology, University of Zurich, Zurich, Switzerland; 4 Department of Social Neuroscience, Max Planck Institute for Human Cognitive and Brain Sciences, Leipzig, Germany; University of Bologna, Italy

## Abstract

People show autonomic responses when they empathize with the suffering of another person. However, little is known about how these autonomic changes are related to prosocial behavior. We measured skin conductance responses (SCRs) and affect ratings in participants while either receiving painful stimulation themselves, or observing pain being inflicted on another person. In a later session, they could prevent the infliction of pain in the other by choosing to endure pain themselves. Our results show that the strength of empathy-related vicarious skin conductance responses predicts later costly helping. Moreover, the higher the match between SCR magnitudes during the observation of pain in others and SCR magnitude during self pain, the more likely a person is to engage in costly helping. We conclude that prosocial motivation is fostered by the strength of the vicarious autonomic response as well as its match with first-hand autonomic experience.

## Introduction

*“No man is an island […], any man's death diminishes me, because I am involved in Mankind” […].* This sentiment of the 17^th^ century clergyman and poet John Donne was confirmed by 20^th^ century psychology research, which shows that humans vicariously experience the suffering of others. Such vicarious experiences have been repeatedly linked to physiological changes such as distinct autonomic nervous system responses when observing another person's suffering [1]–[8]. Most previous studies focused on the conditions modulating vicarious autonomic responses [6]–[8], and on how vicarious autonomic responses are related to autonomic responses experienced in oneself [9]–[11]. One interesting finding was that increased linkage of skin conductance responses between partners is associated with greater accuracy of rating one's partner's affect [10].

In line with these findings, social neuroscience studies demonstrated that sharing the emotions of others recruits neural systems associated with experiencing that emotion oneself [12]–[14]. The strength of such shared neural responses between self and other can be modulated by various factors [15 for review] and, recently, has been linked to individual differences in helping behavior [16], [17].

However, little is known about the link between individual differences in vicarious autonomic responses and the propensity to engage in costly helping. The few previous studies which included measures of autonomic responses and prosocial behavior focused mainly on group differences rather than on predicting individual differences in helping behavior [1], [3], [4]. For example, Eisenberg and colleagues reported that the group average of heart rate deceleration is stronger in sub-samples with prosocial tendencies than in subsamples with no intention to help [1], [3]. Krebs (1975) showed stronger group-averaged autonomic responses, empathic affect and willingness to help in a sample observing pain or reward in a similar person than in a sample observing a dissimilar person [4]. However the link between individual differences in vicarious autonomic responses and individual differences in empathic affect and willingness to help was not assessed.

Here, we investigated whether individual differences in people's skin conductance responses (SCRs) measured when observing another person's pain predict individual differences in later costly helping. Based on previous behavioral studies suggesting a link between empathy and prosocial motivation [18], we postulated that individual differences in the magnitude of empathy-related SCRs when seeing another person in pain would predict individual differences in subsequent costly helping toward that person. Moreover, shared network accounts of empathy propose that understanding the feelings of others relies on the activation of representations which underlie the processing of our own feelings [19], [ 20 for review]. Inspired by this assumption, we investigated how the match in SCR magnitude when experiencing pain in oneself and when observing another person's pain affects later costly helping.

To test these assumptions, we performed an experiment consisting of two Sessions. In Session 1, we collected SCRs and affect ratings while a female participant either received painful or non-painful stimulation herself (conditions Self_Pain and Self_No pain), or while observing another person (a female confederate) receiving painful or non-painful stimulation (conditions Other_Pain and Other_No pain). In a separate Session 2, the participant was not receiving painful stimulation herself, but had to choose from one of three possible options related to impending painful stimulation of the confederate. One option was to volunteer to receive stimulation instead of the confederate. This option represented costly helping as it resulted in pain for the helper herself. The second option was not to help, but to watch a video while pain was delivered to the other. As this distracted participants from watching the pain delivery, it offered an attractive alternative to helping. The third option was not to help, but to watch the other person receive pain in the same way as in Session 1.

## Materials and Methods

### Participants and confederate

Twenty female participants (age: *M* = 24.4 years, *SE* = 0.5) took part in the study, receiving payment for their participation. We recruited only women to avoid variation in emotional and autonomic reactivity due to gender differences. The confederate was a female student unknown to all participants. The study was approved by the University of Zurich ethics committee and performed in agreement with the Declaration of Helsinki. All participants signed informed consent, and could withdraw participation at any point.

### Experimental set up

Participants were informed that they would take part in two sessions – one in which both of them would receive painful and non-painful stimulation and one in which only one of them would receive such stimulation. After giving instructions to Session 1, the experimenter explained that the person who would not receive pain in Session 2 was to be determined by chance (viz., the person who drew the shortest match). Holding up two partially concealed matches, the experimenter made sure that the confederate always drew first and selected the long match, leaving the short one to the participant. Moreover, participants were told that they were going to be paid in different rooms and thus could not meet after the experiment. This measure was taken to ensure that participants' behavior was not driven by expected social feedback, for example fear of retaliation, at the end of the experiment. Participant and confederate were seated opposite to each other on a table, separated by a non-transparent black curtain with a cut-out enabling them to see each other's hands with the pain electrode attached. Each person had a separate screen, keyboard and computer on her side of the curtain, as well as the necessary equipment to measure SCRs. However, in reality only the responses of the participant were recorded. Both wore head phones throughout the experiment.

### Visual stimulation

The pain level (pain or no pain) and the recipient (self or other) were indicated with flash-shaped cues, displayed in light blue/green (no pain) or dark blue/green (high pain), left (stimulation of participant) or right (stimulation of confederate) of a fixation cross placed in the center of the screen. Colors were counter-balanced across participants, but kept constant across sessions for each participant. From the onset until the offset of stimulation, the color of the flash turned into white-orange for painful stimulation, and into light grey for non-painful stimulation.

### Pain stimulation

Pain was delivered with a custom made pain stimulator [21], using electrical stimulation (monopolar, monophasic, pulse width: 500 ms; frequency: 30 Hz; duration: 500 ms). Individual levels of pain stimulation were calibrated using a standard procedure. Briefly, participant and confederate were individually asked to imagine a scale from 1–10, with “one” corresponding to a non-painful sensation and “ten” corresponding to extreme, unbearable pain. For the experiment, we used the level subjectively rated as “one” in the no pain condition, and the level rated as “eight” (tolerable yet strong pain) in the high pain condition. The individual pain levels were again tested in a short practice block (12 trials) prior to the main experiment, in which participants rated their affect before receiving pain or no pain on a visual analogue scale.

### Visual analogue scale

Participants responded to the question “How do you feel?” (in German) by moving a cursor between two endpoints labeled as “very bad” and “very good”, respectively, and a middle point labeled as “neutral”. For numerical analyses, responses were coded from 0 (most negative feeling) to 100 (most positive feeling).

### Psychophysiological measurements

A PowerLab 26T amplifier was used to record electrodermal responses, using a GSR Amp unit and a pair of finger electrodes (ML116F) attached to the participants left middle and ring finger, using dedicated Velcro straps and a bipolar signal amplification setup. Hands had been washed using soap without detergents before the experiment, and thoroughly air dried. Stable recordings were ensured by waiting for signal stabilization before starting the experiment. LabChart (v. 5.5) software was used for recordings, with the recording range set to 40 µS and using initial baseline correction (“subject zeroing”) to subtract the participant's absolute level of electrodermal activity from all recordings (all specs for devices and electrodes from ADInstruments Inc., Sydney, Australia).

### Session 1

Session 1 investigated affect ratings and electrodermal responses to painful and non-painful stimulation of the self or the other (confederate). After a fixation period of 8000 ms, each trial started with a cue indicating the target and the type of stimulation (self or other; painful or non-painful). After a variable delay (mean = 6000 ms, range 4000 ms to 12000 ms; mean and variance of the delay were kept constant across the four conditions, i.e., painful/non-painful×self/other), the cue changed its color, synchronized with delivery of the stimulation, but lasting 500 ms longer. Then the visual analogue scale was shown for 4000 ms. Participants rated how they felt when receiving stimulation themselves, or how they felt when observing the other person receiving stimulation. Each session consisted of sixteen blocks with five stimuli (pain or no pain). Half of these blocks included stimulation of the self, half of them stimulation of the other, with block order and stimulus type being pseudo-randomly permuted (repetitions of block type were limited to one, and stimulus type repetitions to three).

### Session 2

The experimental setup (position of the confederates, pain electrodes) and the overall design (cues, timing) were similar to Session 1. The main difference was that now only the confederate was selected to receive painful or non-painful stimulation, while the participant was asked to choose between three decision options: (1) to help by taking the other person's stimulation on her own hand (Help), (2) not to help, but to watch the other person receive stimulation (Watch Stimulation), or (3) not to help, but to watch a video while the confederate was receiving stimulation (Watch Video). After a cue indicated the type of stimulation that the confederate would receive (i.e., painful or non-painful), the decision of the participant was prompted by a three-key display shown for 4000 ms. Participants made their choice by pressing the leftwards (Help), upwards (Watch Video) or rightwards (Watch Stimulation) arrow key of the keyboard. The decision to help was followed by a cue indicating upcoming delivery of stimulation to the self, which changed its color when stimulation was delivered. The decision to watch a video was followed by a 7 s video, showing pleasant landscapes (without any social content). The participant knew that while she was watching the video, the other was receiving the type of stimulation that had been indicated before the decision screen, though without knowing the exact time of delivery. The decision to watch the other receive stimulation was followed by the cue indicating the level of stimulation the other was about to receive, which changed color when stimulation was delivered. Since we were mostly interested in costly helping, 30 of the 50 trials consisted of painful stimuli, and 10 of non-painful ones. Moreover, there were 10 “computer-generated” mock trials, in which the word “computer” appeared on the screen of the participant, while the other saw the same sequence of events on the screen as in all other trials. In these trials, so the participant and the confederate were told, the computer assigned painful or non painful shocks to the confederate. This manipulation ensured that a) the other could not know whether the stimulation she received resulted from the decision of the participant or had been randomly assigned by the computer and b) that the participant was aware of this, preventing that helping was motivated by reputation concerns. The non-painful stimulation trials were included in Session 2 because we expected it to be irritating and implausible for the participant to see the other person invariably facing highly painful shocks. Mock trials and trials with non-painful stimulation were not included in the analyses. Trial sequence was pseudo-randomized, with a maximum repetition of four painful trials.

Not all decision options were used by all participants. This resulted in a large number of empty decision cells across participants, which would have considerably reduced the statistical power of the SCR analyses in Session 2. Taking this into account, and given that the focus of our study was to predict helping behavior in Session 2 from SCRs in the independent Session 1, we did not analyze the SCRs from Session 2.

### Data analysis

SCR analysis was performed using custom-made Python scripts (www.python.org; www.scipy.org). First, the peak-to-peak difference in conductance was extracted for all SCRs. SCR onset times were determined as positively sloped zero-crossings of the first derivative. Overlapping SCRs were separated at minima in the first derivative and entered the analysis as separate responses. Responses <0.005 µS were discarded, because they are likely to reflect measurement noise. Individual SCRs were log-transformed to correct for skewness, and range-corrected by each participant's maximum response [22]; SCRs are reported as units of range-corrected log_e_(µS).

Based on a moving window average of SCRs, we determined 1.5–5 s after cue onset as the time window for statistical analysis (**Figure S1A**). Trials with delay periods lower than 5 s were excluded to avoid contamination from artefacts caused by the pain stimulator. The SCR values entering the analyses are individual averages across all trials from SCRs summed for each trial in this time window. To account for habituation effects (documented in **Figure S1B**) we restricted all SCR analyses to trials of the first half of Session 1, of which average responses per participant and per condition were computed.

Our main goal was to assess the link between the strength of vicarious SCRs in Session 1 and costly helping in Session 2. Accordingly, we analyzed participants' SCRs when observing the other's pain in Session 1, and correlated individual differences in SCR magnitudes with individual differences in the percentage of helping decisions in Session 2. We also analyzed the correlation between participants' SCR magnitudes and their average affect ratings when observing the other's pain in Session 1. To assess whether the difference in SCR magnitude when experiencing self pain and observing the other's pain has an impact on costly helping, we calculated a Self-Other-SCR-Difference score for each participant, and correlated it with the percentage of helping in Session 2. This score was calculated as the absolute value of the difference in magnitude between the SCRs during self pain and the SCRs when observing the other in pain (|(SCR Self_Pain – SCR Other_Pain)|). In addition, we conducted a hierarchical multiple regression analysis [23] to test the contributions of SCR Other_ Pain, SCR Self_ Pain and the Self-Other-SCR-Difference score to explaining variance in helping. Given that our main assumption postulated a correlation between SCR Other_ Pain and helping, in a first step we included SCR Other_ Pain, using forced entry. In a second step, we added the variables SCR Self_Pain, and the Self-Other-SCR-Difference score to explore whether they explained additional variance. Since we did not have a priori assumptions about the impact of these two variables on helping which could determine the order of their entry, we used a stepwise procedure. All variables were checked for outliers, i.e., values with a deviation of more than 3*SD* from the mean. We report Pearson correlation coefficients with *p*≤0.05. As most of our analyses tested directed hypotheses, we report one-tailed *p*-values, unless indicated otherwise.

Before conducting the correlation analyses, participants' average SCRs and affect rating scores of the Self_Pain and Self_No pain, Other_Pain and Other_No pain conditions were submitted to paired t-tests to test general effects of the experimental manipulations. Moreover, we tested for habituation effects in affect ratings of Session 1 and behavioral decisions of Session 2 by comparing the results of the first with those of the second half of the sessions.

## Results

### Effects of pain manipulation

Confirming the success of the pain manipulation in the self and other-related conditions, SCRs were significantly stronger when participants received painful (*M* = 0.53, *SE* = 0.6), as compared to non-painful stimulation (*M* = 0.25, *SE* = 0.03), *t*(19) = 5.31, *p*<0.001, *η_p_^2^* = 0.60, and when they observed painful stimulation of the other (*M* = 0.23, *SE* = 0.05) as compared to non-painful stimulation (*M* = 0.1, *SE* = 0.02), *t*(19) = 2.78, *p* = 0.006, *η_p_^2^* = 0.29. In line with the SCR results, mean affect ratings reflected more negative affect in the Self_Pain (*M* = 25.21, *SE* = 2.81) than in the Self_No pain condition (*M* = 53.89, *SE* = 2.82, *t*(19) = 10.21, *p*<0.001, *η_p_^2^* = 0.85), and when observing painful stimulation (*M* = 39.92, SE = 2.77) as compared to non-painful stimulation in the other (*M* = 57.04, *SE* = 2.79), *t*(19) = 3.93, *p*<0.001, *η_p_^2^* = 0.45). The computation of ANOVAs with the factors pain (pain/no pain) and time (first half/second half), calculated separately for affect ratings in the self and the other-related conditions in Session 1, showed no significant effects of time. This indicates that affect ratings were not influenced by habituation (self/other condition, factor pain, *F(*1,19) = 104.4, *p*<0.001, *η_p_^2^* = 0.85/*F*(1,19) = 45.1, *p*<0.001, *η_p_^2^* = 0.70; factor time, *F*(1,19) = 0.04, *p* = 0.83, *η_p_^2^* = 0.002/*F*(1,19) = 0.06, *p* = 0.81, *η_p_^2^* = 0.003; interaction pain×time, *F*(1,19) = 0.63, *p* = 0.43, *η_p_^2^* = 0.032/*F*(1,19) = 0.16, *p* = 0.69, *η_p_^2^* = 0.008).

### Decisions in Session 2

On average, the costly helping option was chosen in 53.75% (*SE* = 5.5), the Watch Video option in 30.5% (*SE* = 6.3), and the Watch Stimulation option in 15.8% (*SE* = 3.8) of all painful trials. However, the latter was chosen by only eight of twenty participants. Therefore, for following analyses we combined Watch Video trials and Watch Stimulation trials, i.e., non-helping trials. Analyses comparing decisions made in the first and the second half of Session 2 showed no significant effects, indicating no habituation and stable decisions across repeated trials (Mann-Whitney test, *U* = 44769.5, *Z* = −0.12, *p* = 0.9).

### Correlation analyses

**Figure 1:**
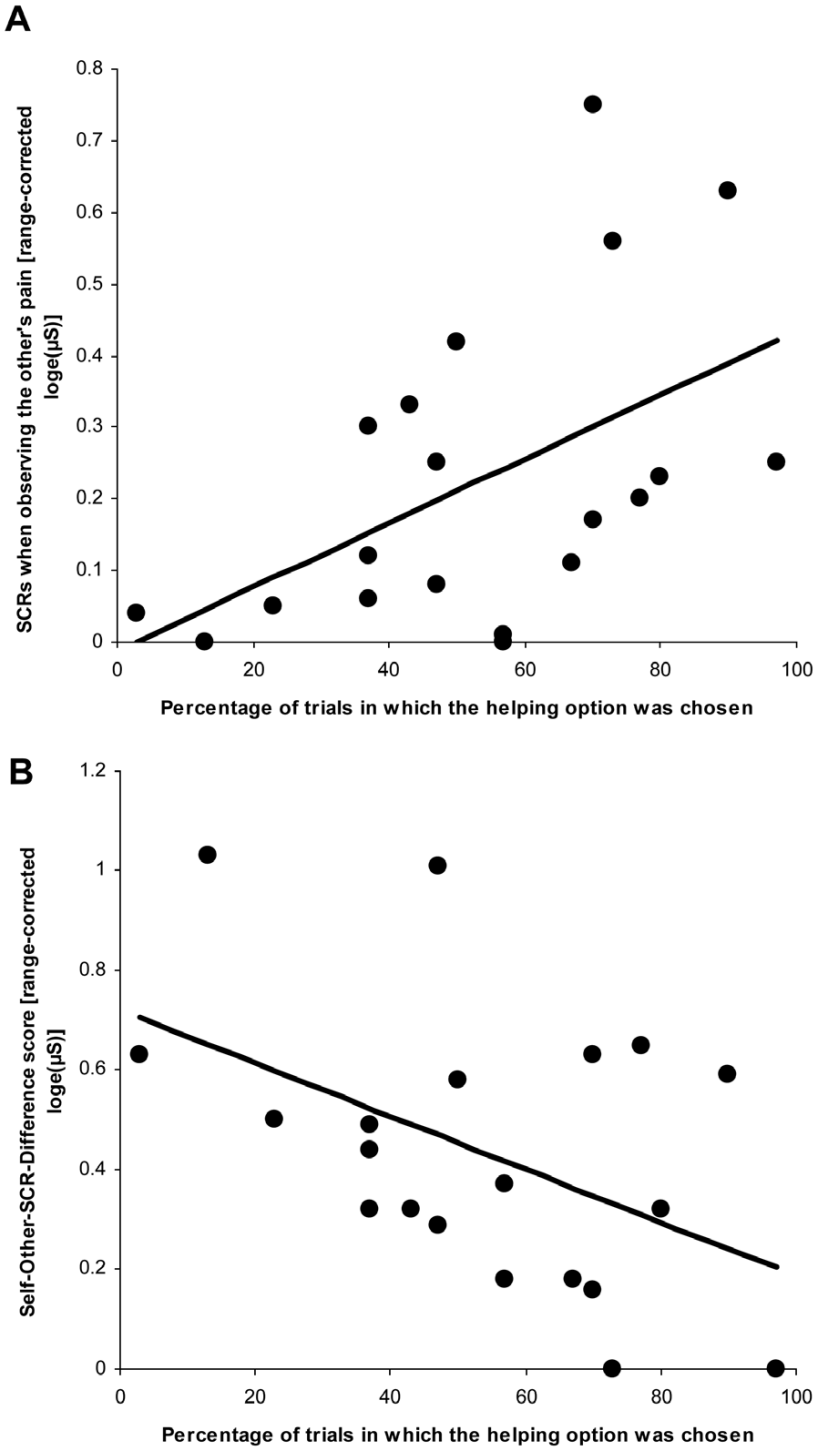
Main results of the correlation analyses. **A**) Significant positive correlation between participants' skin conductance responses (SCRs) when seeing the other person in pain, and percentage of trials in which they chose the costly helping option (out of a total of 30 trials). **B**) Significant negative correlation between the score measuring the difference between the SCR to self pain and when observing the other's pain (|(SCR_self pain – SCR_other pain)|), and the percentage of trials in which they chose the costly helping option (out of a total of 30 trials).

As predicted, we obtained a positive correlation between individual magnitudes of SCR Other_Pain in Session 1 and the percentage of trials in which participants chose to help in Session 2, *r*(20) = 0.51, *p* = 0.01 (**Figure 1A**). Furthermore, we found a negative correlation between SCR Other_Pain and the decision not to help, i.e., either to watch a video or to watch the other receiving painful stimulation, *r*(20) = −0.52, *p* = 0.02.

Correlating affect ratings and SCRs in Session 1 in the Other_Pain condition yielded a significant negative correlation *r*(20) = −0.53, *p* = 0.008. Since lower affect ratings reflect more negative feelings, the negative correlation indicates higher SCR magnitude with stronger negative vicarious affect. The correlation between affect ratings and SCR in the Self_Pain condition showed a trend towards significance, *r*(20) = −0.37, *p* = 0.053.

Finally, we found a negative relationship between the frequency of helping and the Self-Other-SCR-Difference score, *r*(20) = −0.47, *p*(two-tailed) = 0.038. This indicates that the smaller the absolute difference of SCR magnitudes during the direct and the vicarious experience of pain, the more often participants helped (**Figure 1B**), and vice versa.

The hierarchical multiple regression analysis showed that SCR Other_Pain alone was a significant predictor for later helping, *F*(1,19) = 6.44, *p* = 0.021; adjusted *R^2^* = 0.22 (**Table 1**; Step 1). Adding the Self-Other-SCR-Difference score resulted in an additional 20.8% of variance being explained, *F*(2,19) = 7.59, *p* = 0.004; adjusted *R^2^* = 0.41, Δ*R^2^* = 0.21, *p(F change)* = 0.019 (**Table 1**; Step 2). The SCR Self_Pain variable did not contribute to the model. The same result was obtained when adding the Self-Other-SCR-Difference score and the SCR Self_Pain in reversed order (Self-Other-SCR-Difference score first; SCR Self_Pain second).

**Table 1:** The unstandardised and standardised regression coefficients for the variables included in the model which best accounted for variance in Helping.

Predictors	B	*SE* b	*B*	*t*-value	*p*-value
Step 1					
Constant	0.4	0.07			
SCR_Other_Pain	0.59	0.23	0.51	2.54	0.011
Step 2					
Constant	0.58	0.09			
SCR_Other_Pain	0.58	0.2	0.5	2.85	0.011*
Self-Other SCR Difference	−0.4	0.16	−0.46	−2.59	0.019*

SCR_Other_Pain = Skin conductance responses when observing the other's pain; Self-Other SCR Difference = absolute difference between skin conductance responses when participants received pain themselves and when observing pain in the other person. b = unstandardized coefficient; *SE* = standard error; *β* = standardized coefficient, providing a measure of the contribution of each variable to the model;

*p-value = two-tailed.

## Discussion

Our study investigated the link between a person's SCRs when observing the suffering of another person, and later costly helping. As a first main result, we found that individual differences in vicarious SCRs predicted later decisions to help at own cost. This finding extends previous studies which investigated differences in average autonomic responses, affect ratings and prosocial tendencies between different groups [1], [4]. Notably, the magnitude of SCRs when observing the other's pain correlated with participants' vicarious affect ratings, suggesting that this autonomic measure reflects the vicarious emotional responses to the other's affective state. Taken together, our findings support the assumption that empathy motivates prosocial behavior, directed toward the goal of increasing the welfare of a person in need [18].

Secondly, our results revealed that the difference between autonomic responses during self-experienced and vicariously experienced pain is a significant predictor for later costly helping: the more similar the magnitudes of self- and other-related SCRs the more likely the participant was to help the other. Previous reports have suggested a link between physiological correlates of self-experienced emotions and vicarious physiological responses to other's emotions. For example, it has been shown that a stronger linkage in skin conductance between partners increased empathic accuracy [10], and that empathy with the pain of others is accompanied by activations in brain regions related to the affective processing of self-experienced pain [13 for review]. Our results substantially extend these findings by showing that the extent to which the vicarious autonomic response resembles the self-related autonomic response in the same situation affects costly helping behavior directed towards the other. Most notably, the hierarchical multiple regression analysis showed that this match between vicarious and self-related autonomic responses explains *additional* variance in later costly helping, i.e., variance that is not explained by vicarious autonomic responses alone. This indicates that costly helping is more likely to occur if the empathy-related autonomic response is not only strong, but in addition resembles the autonomic response the helper shows when experiencing the same situation.

Existing psychological models have emphasized the importance of feeling *for* the other, i.e., the strength of emotions related to empathic concern, as a predictor for helping behavior [18]. Our findings add a novel aspect to these models by showing that the match between first- and second-person autonomic experiences, i.e., feeling *as* the other person, is equally important for a person's willingness to expose themselves to aversive stimulation in order to alleviate the suffering of others.

## Supporting Information

Figure S1 **Time course of the skin conductance response for all conditions of Session 1.** **A**) The plot indicates the mean ±2 SEM of a moving window average (1 s Blackman window), and is based on the first half of the trials. The blue vertical lines indicate the time window of 1.5–5 s after cue onset used for the statistical analyses. **B**) Skin conductance responses of all conditions in the first and the second half of Session 1. The results indicate significant habituation of skin conductance responses in the second half of the session for all conditions.
(TIF)
