# Supplementary figures and images for: Skin Conductance Response to the Pain of Others Predicts Later Costly Helping
Source: PLoS One. 2011 Aug 3;6(8):e22759. doi: 10.1371/journal.pone.0022759 (PMC3149614; doi:10.1371/journal.pone.0022759)

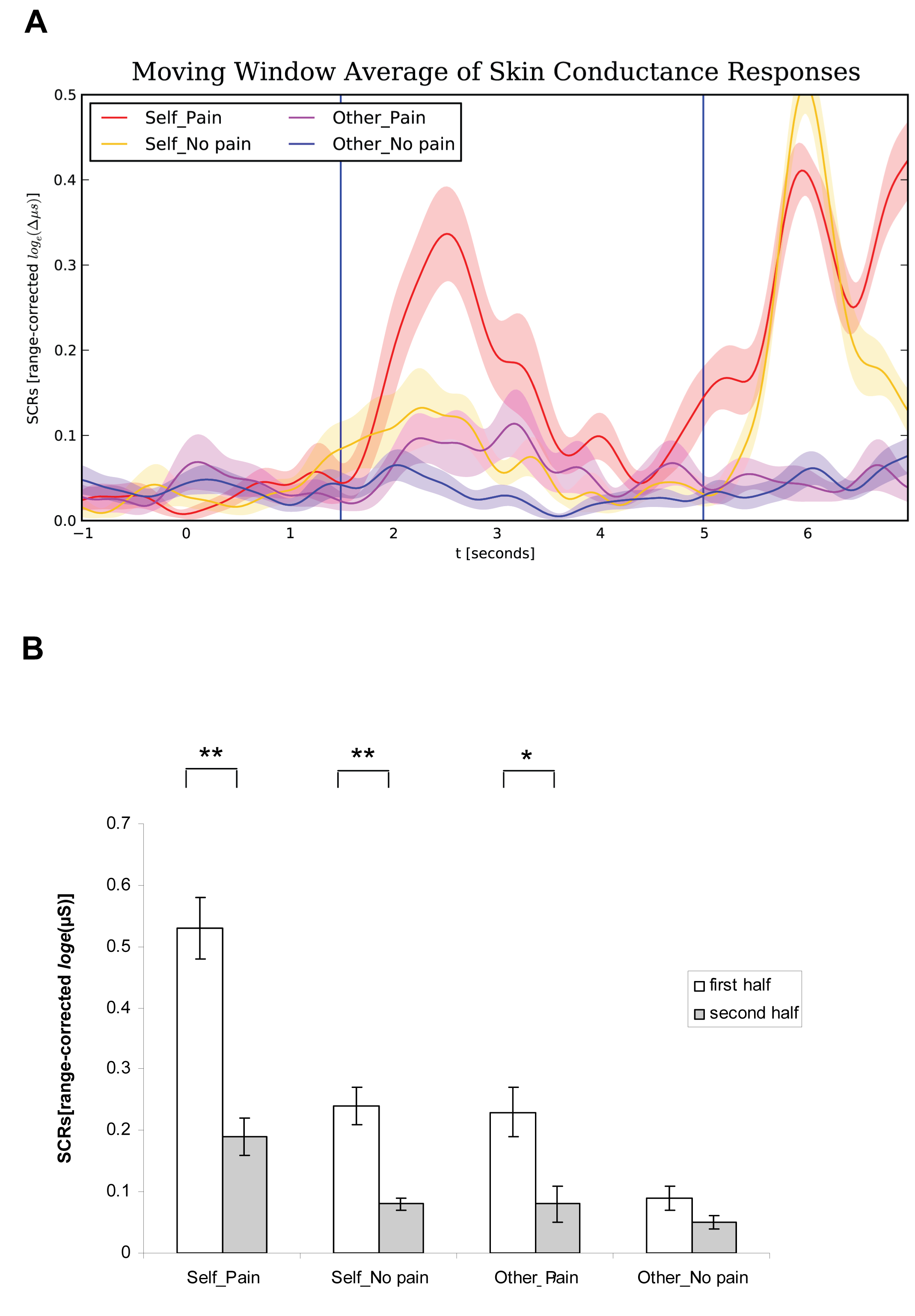

Supplement: Figure S1 — Time course of the skin conductance response for all conditions of Session 1. A) The plot indicates the mean ±2 SEM of a moving window average (1 s Blackman window), and is based on the first half of the trials. The blue vertical lines indicate the time window of 1.5–5 s after cue onset used for the statistical analyses. B) Skin conductance responses of all conditions in the first and the second half of Session 1. The results indicate significant habituation of skin conductance responses in the second half of the session for all conditions. (TIF) [file pone.0022759.s001.tif]
